# High-fidelity and polarization-insensitive universal photonic processors fabricated by femtosecond laser writing

**DOI:** 10.1515/nanoph-2023-0636

**Published:** 2024-01-16

**Authors:** Ciro Pentangelo, Niki Di Giano, Simone Piacentini, Riccardo Arpe, Francesco Ceccarelli, Andrea Crespi, Roberto Osellame

**Affiliations:** Dipartimento di Fisica, Politecnico di Milano, Milano, Italy; Istituto di Fotonica e Nanotecnologie, Consiglio Nazionale delle Ricerche (IFN-CNR), Milano, Italy

**Keywords:** femtosecond laser writing, universal photonic processor, integrated photonics

## Abstract

Universal photonic processors (UPPs) are fully programmable photonic integrated circuits that are key components in quantum photonics. With this work, we present a novel platform for the realization of low-loss, low-power, and high-fidelity UPPs based on femtosecond laser writing (FLW) and compatible with a large wavelength spectrum. In fact, we demonstrate different UPPs, tailored for operation at 785 nm and 1550 nm, providing similar high-level performances. Moreover, we show that standard calibration techniques applied to FLW-UPPs result in Haar random polarization-insensitive photonic transformations implemented with average amplitude fidelity as high as 0.9979 at 785 nm (0.9970 at 1550 nm), with the possibility of increasing the fidelity over 0.9990 thanks to novel optimization algorithms. Besides being the first demonstrations of polarization-insensitive UPPs, these devices show the highest level of control and reconfigurability ever reported for a FLW circuit. These qualities will be greatly beneficial to applications in quantum information processing.

## 1 Introduction

Quantum information processing is a rapidly advancing field that aims at harnessing the unique properties of quantum mechanics, such as superposition and entanglement, to perform computation and communication tasks that are impossible or difficult using classical methods. Photonics offers several advantages over other approaches in this framework [1]. Photons are highly stable and can travel long distances without being absorbed or suffering decoherence even at room temperature. Their flying nature makes them also the most natural way to transfer quantum information. Furthermore, interest in this approach has recently increased after the experimental demonstrations of quantum supremacy in photonic systems [2], [3].

One promising and scalable approach to implement quantum computing and quantum communication protocols is through the use of photonic integrated circuits (PICs) [4]. Integrated photonics allows to miniaturize optical components and integrate them on the same substrate, leading to high scalability and integration density while guaranteeing an intrinsic optical stability even among a large number of components. Programmability of the PICs operation is typically achieved by actively controlling the phase shifts [5]. The simplest and most widely implemented form of phase shifters are thermal phase shifters, which exploit the thermo-optic effect by dissipating electrical power into heat, reversibly modifying the waveguide refractive index.

The simplest fully programmable PIC is the Mach–Zehnder interferometer (MZI), which is a 2-port circuit featuring two balanced directional couplers and two phase shifters. This device can implement any unitary transformation between the input and output modes. The generalization to an *N*-mode circuit can be done by employing a mesh of MZIs in triangular [6] or rectangular [7] configuration, thus obtaining a circuit that is able to perform any unitary transformation in *U*(*N*). These universal photonic processors (UPPs) are key components for quantum information processing and have been already demonstrated in various photonic platforms and materials [8]–[18]. Among them, femtosecond laser writing (FLW) of waveguides in silicate glass [19] features low insertion losses and low birefringence over a wide wavelength spectrum ranging from the visible to the near-infrared. This fabrication technique is quite versatile: it not only allows for cost-effective and rapid prototyping of PICs but also enables to ablate the substrate with femtosecond pulses and thus cut out microstructures. The micro-structuring of the substrate allowed by FLW can be used to fabricate thermal isolation structures [20] that, in conjunction with thermal phase shifters, significantly reduce their power dissipation and crosstalk of orders of magnitude.

In this work, we demonstrate the potential of the FLW platform by fabricating and calibrating two 6-mode UPPs operating at 785 nm and 1550 nm, respectively. These circuits feature insertion losses at 785 nm (1550 nm) lower than 3 dB (2.5 dB), average 2*π* power dissipation per phase shifter as low as 39 mW (63 mW), and are able to implement unitary transformations with an average amplitude fidelity of 0.9979 (0.9970), which can increase over 0.9990 by exploiting optimization algorithms as we show experimentally in this work, and which does not depend on the H/V polarization state of the input light. These devices are among the few examples of UPPs currently reported in the literature showing such a high level of control and reconfiguration accuracy over a wide set of implemented transformations and, to the best of our knowledge, the first processors featuring a polarization-insensitive behavior.

## 2 Design and fabrication

**Figure 1:**
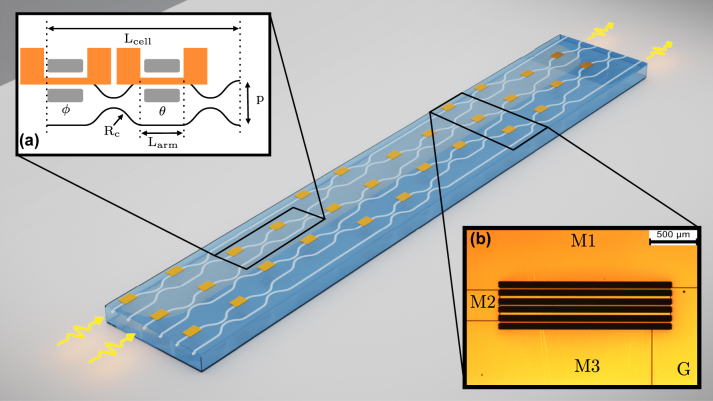
3D rendering of the UPP. Inset (a) shows the schematic layout of an individual MZI of the device. The internal and external phases of the MZI are labeled *θ* and *ϕ*, respectively. The gray areas represent trenches. Inset (b) is a microscope picture of UPP A comprising a column of three thermal shifters, where it is possible to see the trench structures (larger rectangular structures), the metal film (orange) and the ablations in the film. M1, M2, and M3 are three contact pads used to independently bias the thermal shifters, while G is their common ground.

Processors at 785 nm and 1550 nm (UPP A and B, respectively, from now on) share the same waveguide layout based on a rectangular mesh [7] of 15 MZI-based unit cells, entailing a total number of 30 thermal shifters (Figure 1). The unit cell reported in [20] is here employed for UPP A and depicted in Figure 1 (inset a). The pitch between adjacent waveguides is *p* = 80 µm. Balanced directional couplers are realized by bending the waveguides with a minimum curvature radius of *R*_*c*_ = 30 mm, while MZI arms (and thermal shifters) are *L*_arm_ = 1.5 mm long. The total length of the cell is *L*_cell_ = 11.4 mm. This results in a chip dimension of 80 × 20 mm including also the fan-in and fan-out sections at each end of the circuit added for compatibility with standard 127 µm fiber arrays. In order to compensate for the longer operating wavelength and keep the same temperature profile [20] for a given phase shift, UPP B instead features longer MZI arms (*L*_arm_ = 3 mm). Constant-temperature scaling allows us to produce devices sharing the same properties in terms of stability, breakdown power, nonlinearity, etc., paying a small price in terms of unit cell length. However, this penalty is partially compensated by employing more confining waveguides featuring a curvature radius of *R*_*c*_ = 15 mm. The reduced radius leads to a total length of the cell *L*_cell_ = 13.2 mm and, as a result, to a chip dimension of 90 × 20 mm.

Fabrication of these devices starts from a 1 mm thin Corning Eagle XG alumino-borosilicate glass substrate. Waveguides are inscribed at a depth of 30 µm from the surface, by multi-scan laser irradiation followed by thermal annealing of the substrate [21]. Waveguide irradiation parameters are optimized for single-mode operation at the respective wavelengths for the two processors. The waveguides have a guided mode (1/*e* ^2^) of 4.5 × 4.7 µm^2^ for UPP A and 8.5 × 9.0 µm^2^ for UPP B. The waveguide propagation losses are 0.2 dB cm^−1^ for UPP A and 0.15 dB cm^−1^ for UPP B. Additional bending losses in the curved segments are about 0.1 dB cm^−1^ for both devices. Thermal isolation trenches are machined by water-assisted laser ablation on each side of the top arm of each MZI both before and after the first directional coupler, where the thermal shifters will be fabricated [20]. All trenches are 300 µm deep, 60 µm wide, and either 1.5 mm or 3 mm long, respectively, for devices A and B. Fabrication of the resistive microheaters of the thermal phase shifters is based on the process reported in [22]. A thin gold layer is deposited on the surface of the device by thermal evaporation and then etched with femtosecond laser pulses so that 10 µm wide microheaters are located on top of the desired MZI arms, while larger contact pads allow for their connection at the sides of the die. A large aspect ratio for the contact pads is required to limit their parasitic series resistance, given that both they and the microheaters are fabricated on the same gold film. Figure 1 (inset b) is a micrograph of UPP A showing a column of three MZI cells, in which it is possible to easily identify trenches, microheaters, and contact pads. After packaging the die on an aluminum heat sink, the thermal shifters are connected to printed circuit boards by means of electrically conductive epoxy glue, allowing easy interfacing with the external electronics. Final resistance values for the microheaters are 111 ± 6 Ω (UPP A) and 215 ± 15 Ω (UPP B). Finally, the input and output ports of the circuits are made available for characterization by standard optical fiber arrays pigtailed with UV-curing glue. The coupling losses can be estimated as 0.5 dB per facet for UPP A and 0.3 dB per facet for UPP B. At the end of this process, total insertion losses of about 3 dB and 2.5 dB are measured for UPP A and B, respectively.

## 3 Modeling and calibration

The transfer matrix of the MZI unit cell reported in Figure 1 (inset a) can be expressed as:

(1) $$U_{\text{MZI}}={\text{e}}^{\text{i}\left(\frac{\theta }{2}+\frac{\pi }{2}\right)}\left[\begin{array}{cc} e^{i\phi }\sin\left(\frac{\theta }{2}\right) & \cos\left(\frac{\theta }{2}\right)\\ e^{i\phi }\cos\left(\frac{\theta }{2}\right) & -\sin\left(\frac{\theta }{2}\right) \end{array}\right],$$

where *ϕ* and *θ* are the phases induced by the external and internal phase shifters, respectively, (see Figure 1, inset a). Assuming to inject light in one input port of this cell, the normalized optical power *P*_out_ measured at the cross output port will depend only on the internal phase *θ* as:

(2) $$P_{\text{out}}=\frac{1+\cos\left(\theta \right)}{2}.$$

**Figure 2:**
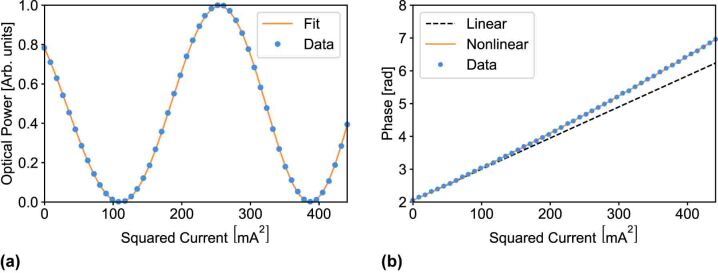
Experimental characterization of individual MZIs on UPP A. (a) Optical power *P*_out_ measured at the cross output as a function of the squared current *I* ^2^ when an internal thermal shifter is actuated. Best fit and experimental dataset are both reported, showing the effectiveness of our model, based on Equations (2) and (3). (b) Phase *θ* as a function of the squared current *I* ^2^ obtained from the dataset reported in (a). The solid orange line represents the best nonlinear (polynomial) fit. The dashed black line represents the expected trend without the second-order term (i.e., *β* = 0 in Equation (3)). Error bars are smaller or comparable to the marker size.

The phase *θ* induced by a thermal shifter can be tuned by controlling either the voltage drop *V* across the microheater or the current *I* flowing through it. In our case, we decided for the latter in order to prevent the nonlinear crosstalk due to pure electrical phenomena [22]. An example of interference measured on an individual MZI is reported in Figure 2a, where the optical power *P*_out_ is reported as a function of the squared current *I* ^2^. Indeed, the phase *θ* induced by each shifter can be expressed as follows:

(3) $$\theta =\theta_{0}+\alpha_{I}I^{2}\left(1+\beta I^{2}\right),$$

where the constant phase term *θ*_0_ is an offset present due to fabrication tolerances, *α*_*I*_ is the tuning coefficient of the thermo-optic process, and *β* is a correction factor needed to take into account that the microheater resistance depends on the temperature. Such a nonlinear effect is highlighted in Figure 2b, where *θ* is reported as a function of the squared current *I* ^2^. In addition, it is also necessary to consider the thermal crosstalk effects. Indeed, the phase induced on the *i*-th MZI in the circuit will be affected by all of the active microheaters and thus:

(4) $$\theta_{i}=\theta_{0,i}+\underset{j}{\sum }\alpha_{ij}I_{j}^{2}\left(1+\beta_{j}I_{j}^{2}\right),$$

where the superposition principle is employed in spite of the presence of the correction term thanks to the fact that the latter depends in first approximation only on the *j*-th shifter. In addition, it is worth noting that the constants *α*_*ij*_ strongly depend on the distance between the *i*-th MZI and *j*-th shifter. Due to the large bending radii (relative to the inter-waveguide pitch) of these circuits, horizontally neighboring MZIs are millimeters apart while vertically neighboring MZIs are 160 µm apart. This means that we can neglect the coefficients *α*_*ij*_ for pairs of MZIs that are not vertically adjacent, leading to a significant simplification of the calibration process and improved control accuracy.

The dataset composed by *θ*_0,*i*_, *α*_*i*,*j*_, and *β*_*j*_ represents the calibration dataset for the internal shifters. In order to retrieve it, coherent light is injected in each individual MZI following a node isolation algorithm [23]. Then, the output optical power dependence on the electrical power is fitted from Equations (2) and (4) in order to obtain all the parameters for individual shifters and pairs connected by crosstalk effects. During this process, internal shifters that are already calibrated are set to obtain behaviors as straight waveguides (*θ* = *π*), crossings (*θ* = 0), or balanced beam splitters (*θ* = *π*/2). By surrounding a yet uncalibrated MZI with fully reflective or fully transmissive paths, it is possible to isolate it and proceed with a clean characterization of the phase shifter.

For external shifters, the procedure follows both the same modeling and measurement strategy. The only difference is the necessity to enclose the phase shifter in larger interferometric rings formed by multiple MZIs [8], [13]. All of these measurements have been automated with custom Python scripts to control the instrumentation involved and fit the parameters. More information about the calibration apparatus is reported in the Supplementary Materials (Section S1).

To set a specific unitary transformation *U* on a UPP, one can use the decomposition reported in [7] to obtain the corresponding set of phases *θ*_*i*_ and *ϕ*_*i*_. Then, it is possible to invert Equation (4) to find the set of currents *I*_*i*_ that implement the desired phases. Since this problem in general does not have a unique solution, we always look for the set of currents *I*_*i*_ that minimizes the total power budget dissipated on chip. With this method, the measured dissipated power was always lower than 1.2 W in UPP A (1.9 W in UPP B). From this calibration procedure, it is already possible to estimate the average 2*π* power dissipation of each thermal phase shifter, which is 39 mW for UPP A and 63 mW for UPP B.

## 4 Experimental results

In this section, we present the experimental results obtained on the two devices by starting with the implementation of the unitary transformations, showing how this implementation can be optimized with numerical methods and concluding by analyzing the effect of the polarization.

### 4.1 Implementation of unitary transformations

The successful calibration of UPPs A and B was verified with the same experimental setup by implementing two types of unitary transformations: switching transformations, where the device acts as an optical switch linking each input with a given output, and Haar random transformations, corresponding to randomly sampled complex unitary matrices. The former only requires the actuation of internal shifters (specifically to either *θ* = 0 or *θ* = *π*) while the latter requires the actuation of both internal and external shifters to arbitrary phase values. Each measurement can be summarized as follows:

1. Sample a random switching or Haar random matrix *U*_out_ ∈ *U*(6).
2. Find the set of phases *θ*_*i*_ and *ϕ*_*i*_ corresponding to *U*_set_ using the decomposition algorithm reported in [7].
3. Employ the calibration data to extract and implement electrical currents corresponding to desired phases.
4. Measure the input–output intensity distribution and reconstruct the amplitudes of the experimental matrix *U*_exp_ [24].
5. Evaluate the implementation quality by the amplitude fidelity metric (with *N* = 6 being the number of modes):
(5) $$F_{\text{ampl}}\left(U_{\text{set}},U_{\exp}\right)=\frac{1}{N}tr\left(\left|U_{\text{set}}^{†}\|U_{\exp}\right|\right).$$

**Figure 3:**
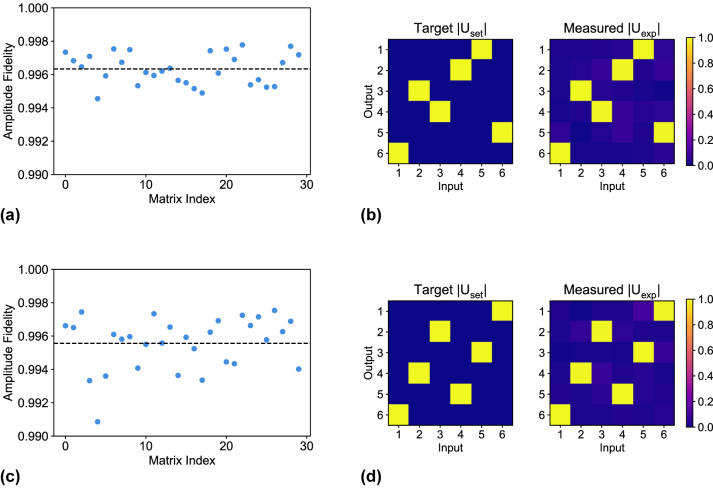
Amplitude fidelity $F_{\text{ampl}}\left(U_{\text{set}},U_{\exp}\right)$ distribution over the 30 randomly chosen switching matrices. (a) Scatter plot of the distribution for UPP A. The average 0.9963 is marked by the dashed line. (b) Example of a switching matrix implementation for UPP A with amplitude fidelity 0.9959. We compare the amplitudes of the target matrix *U*_set_ versus the amplitudes of the measured matrix *U*_exp_. (c) Scatter plot of the distribution for UPP B. The average 0.9956 is marked by the dashed line. (d) Example of a switching matrix implementation for UPP B with amplitude fidelity 0.9960. We compare the amplitudes of the target matrix *U*_set_ versus the amplitudes of the measured matrix *U*_exp_.

**Figure 4:**
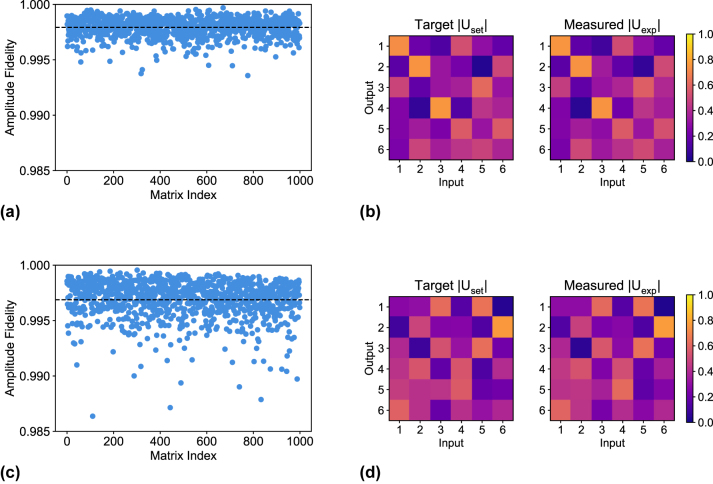
Amplitude fidelity $F_{\text{ampl}}\left(U_{\text{set}},U_{\exp}\right)$ distribution over the 1000 Haar random unitary matrices. (a) Scatter plot of the distribution for UPP A. The average 0.9979 is marked by the dashed line. (b) Example of a unitary matrix implementation for UPP A with amplitude fidelity 0.9975. We compare the amplitudes of the target matrix *U*_set_ versus the amplitudes of the measured matrix *U*_exp_. (c) Scatter plot of the distribution for UPP B. The average 0.9970 is marked by the dashed line. (d) Example of a unitary matrix implementation for UPP B with amplitude fidelity 0.9964. We compare the amplitudes of the target matrix *U*_set_ versus the amplitudes of the measured matrix *U*_exp_.

A total of 30 switching unitaries and 1000 Haar random unitaries were implemented on each UPP and the results are summarized in Figures 3 and 4, respectively.

The amplitude fidelity for the 30 measured switching unitaries is distributed with $F_{\text{ampl}}=\mu \pm \sigma =0.9963\pm 0.0009$ for UPP A (Figure 3a) and 0.9956 ± 0.0016 for UPP B (Figure 3c). An example of implementation is reported in Figure 3b and d, where we compare the amplitudes of the target matrix *U*_set_ with the reconstructed amplitudes of *U*_exp_ and we achieve an amplitude fidelity of 0.9959 (UPP A) and 0.9960 (UPP B). These excellent results not only demonstrate the high accuracy that our calibration protocol can reach on the internal phases but also that the FLW process is able to achieve remarkable accuracy and reproducibility in the implementation of directional couplers with the required splitting ratio.

The amplitude fidelity for the 1000 measured Haar random unitaries is distributed with $F_{\text{ampl}}=\mu \pm \sigma =0.9979\pm 0.0009$ for UPP A (Figure 4a) and 0.9970 ± 0.0017 for UPP B (Figure 4c). An example of implementation is reported in Figure 4b and d, where we compare the amplitudes of the target matrix *U*_set_ with the reconstructed amplitudes of *U*_exp_ and we achieve an amplitude fidelity of 0.9975 (UPP A) and 0.9964 (UPP B).

These results demonstrate that the high calibration accuracy reached for the internal shifters was successfully extended also to the external shifters. Universal reconfiguration and high fidelity control is thus demonstrated for both UPPs. The nonzero residual amplitude infidelity, that is $1-F_{\text{ampl}}$ , is in large part to be ascribed to an imperfect calibration of the MZI mesh’s parameters, as we will demonstrate in the next section by optimizing individual matrices.

### 4.2 Fidelity improvement via single unitary optimization

After successfully demonstrating the implementation of high-fidelity Haar random unitary transformations on both UPPs, we aim at evaluating whether the employed calibration is indeed reaching the limit in terms of accuracy with which our circuits can implement a given matrix. Namely, we tried to improve the implementation of specific transformations by optimizing the electrical currents used to actuate the microheaters. More in detail, the Nelder–Mead algorithm [25] was employed using the amplitude infidelity $1-F_{\text{ampl}}$ as a loss function and the phases set on all phase shifts as variables to optimize. The starting point for the optimization is the set of phases obtained for the target unitary with the decomposition algorithm discussed in the previous section [7]. After each step of the optimization algorithm, the new phases are converted to electrical currents by using the calibration data, the microheaters are actuated, and a new amplitude fidelity is computed from the measurement, which is fed back to the optimizer.

**Figure 5:**
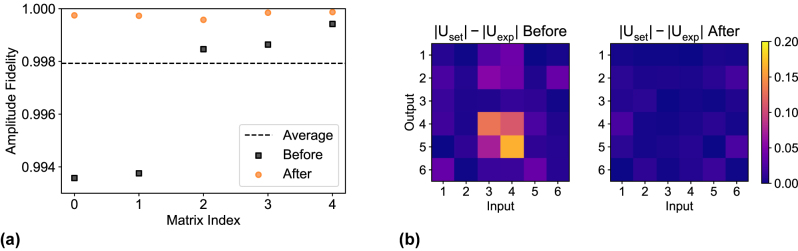
Amplitude fidelity $F_{\text{ampl}}\left(U_{\text{set}},U_{\exp}\right)$ improvement of Haar random matrix implementation through Nelder–Mead algorithm on UPP A. (a) Five unitary matrices (black squares) that were chosen for the optimization and their improved implementation after the optimization process (orange circles). The dashed line is the average fidelity of UPP A over the set of Haar random unitaries as in Figure 4a. (b) Difference between the amplitudes of *U*_set_ and *U*_exp_ before and after the optimization. This particular matrix was optimized from an amplitude fidelity of 0.9936 to 0.9997.

This procedure was applied to 5 of the 1000 Haar random unitaries measured originally on UPP A, selecting some with high, low, or average amplitude fidelity. The results are shown in Figure 5a, where it is clear that even unitaries that were originally measured with high amplitude fidelity can be improved over 0.9995, well above the average for UPP A. A visual comparison between the errors obtained before and after the optimization of a single unitary transformation is shown in Figure 5b, where the amplitude fidelity increased from 0.9936 up to 0.9997. The algorithm was set to run for at most 500 iterations, and all five matrices have hit this stopping condition after 11–12 h. This threshold has been chosen to keep the optimization time reasonable, but it still provides a substantial increase over the original fidelity. These results indicate that it is possible to optimize specific unitary transformations in case higher fidelity is required. In addition, we tried implementing the same unitary repeatedly on the circuit. Over 100 iterations, the average amplitude fidelity between any two measurements of the same unitary transformation is about 0.9998 with this experimental setup. The values reported for the optimized matrices are very close to this limit and, therefore, the current optimization is already the best that we can currently verify. A further optimization will be possible in the future by improving the experimental reproducibility. This result demonstrates that, although the initial calibration alone is not reaching the limit of accuracy given by the experimental apparatus, the maximum fidelity is not currently set by intrinsic limitations of our fabrication process.

### 4.3 Polarization measurement

In all former measurements, the polarization state of light was not controlled. The light polarization at the input of the UPP is determined by the polarization state of light at the output of the laser source and by the action of all the optical elements of the experimental setup. In particular, optical fibers rotate the polarization of light. In performing subsequent measurements, drifts may even have occurred. In the following, we will refer to this as “arbitrary polarization.” We now show additional measurements gauging the variation in the performance of UPP A when using an input state of light that has been accurately set as horizontal (H) or vertical (V). The characterization setup for this experiment is the same used before, with the addition of polarizers and waveplates to arbitrary set the polarization state of the coherent light used for the measurements. In particular, polarizers were set after the laser and in front of the detectors while the waveplates were set between the first polarizer and the device. A complete description of the experimental setup and methods used for this experiment is reported in the Supplementary Materials (Section S1).

As a first step, we sampled a set of 50 Haar random unitary matrices and a randomly chosen set of six switching matrices. Then, we implemented each transformation again, measuring the corresponding input–output intensity distribution with controlled H or V polarized light, thus reconstructing the amplitudes of the experimental matrices *U*_exp,__H_ and *U*_exp,__V_. To better discuss how the implementation depends on the H/V polarization, we show these data here in two different ways.

**Figure 6:**
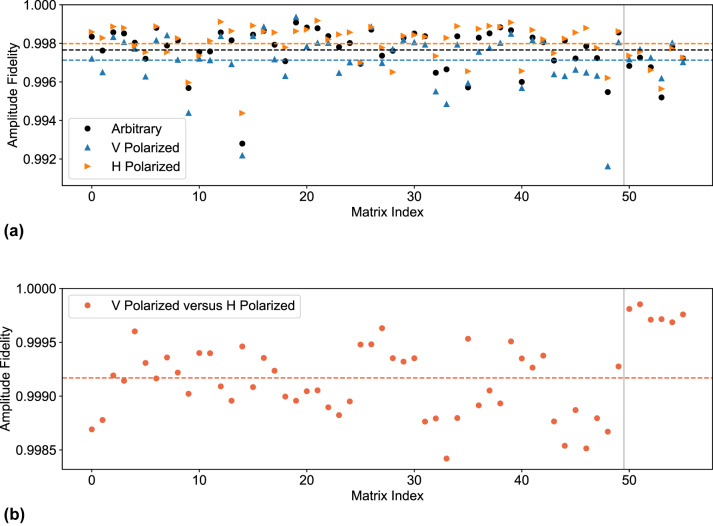
Amplitude fidelity $F_{\text{ampl}}$ distribution with different polarization states on UPP A. For all these plots, the vertical line separates the set of 50 random Haar matrices from the six switching matrices. (a) Scatter plot of the amplitude fidelity $F_{\text{ampl}}\left(U_{\text{set}},U_{\exp}\right)$ where the experimental matrix *U*_exp_ was measured for arbitrary as well as V and H polarized light. The averages 0.9978, 0.9971, and 0.9980 are marked by the black, blue, and orange dashed lines for the three polarization states, respectively. (b) Scatter plot of the amplitude fidelity $F_{\text{ampl}}\left(U_{\text{exp, V}},U_{\text{exp, H}}\right)$ . The average 0.9992 is marked by the dashed line.

Figure 6a shows the amplitude fidelity of the measured matrix calculated against the target matrix *U*_set_ for all three cases: arbitrary, V, and H polarization. The graph shows that the H polarization state performs slightly better on average than the other two, with the V state being the worst overall. Nevertheless, no matrix implementation shows an amplitude fidelity lower than 0.9910 and the average values are 0.9971 for the V polarization and 0.9980 for the H one. This is true not only for the Haar random matrices but also for the switching transformations, providing an additional demonstration of the high polarization insensitivity of the directional couplers.

Then, Figure 6b shows how similar the two matrices *U*_exp,__V_ and *U*_exp,__H_ are by reporting the amplitude fidelity calculated between the two. The average value is 0.9992 and no pair below 0.9984 is reported. Again, it is worth noting that these amplitude fidelities were very close to the experimental limit of our characterization setup, which means that even though the polarization definitely plays a role in the correct implementation of the matrices, it does not have as much of an impact for the purposes of implementation as the calibration and operation of the chip.

## 5 Discussion

In this work, we evaluated the transformations implemented by our UPPs with classical light and intensity measurements, thus reconstructing only the amplitudes of the complex matrix *U*_exp_ representing each transformation. Being largely employed in the literature for the benchmarking of UPPs [9]–[11], [17], [18], we selected the amplitude fidelity (see Equation (5)) as the figure of merit to measure the accuracy reached by our devices in order to guarantee an easy comparison with the literature. However, this topic deserves a deeper discussion.

### 5.1 Amplitude fidelity

For the sake of clarity, let us start by reporting again the definition of the amplitude fidelity $F_{\text{ampl}}$ for the case of two generic unitary matrices *U* = {*u*_*ij*_} and *V* = {*v*_*ij*_}:

(6) $$F_{\text{ampl}}\left(U,V\right)=\frac{1}{N}tr\left(\left|U^{†}||V\right|\right)=\frac{1}{N}\underset{i,j}{\sum }|u_{ij}v_{ij}|.$$

Being an average over *N* scalar products, the amplitude fidelity is a normalized measure of how similar the amplitudes of the two matrices *U* and *V* are. Indeed, the amplitude fidelity is equal to 1 if and only if |*U*| = |*V*|, it is always included in the interval [0,1] and it is directly linked to the amplitude variation matrix |*U*| − |*V*| = {|*u*_*ij*_| − |*v*_*ij*_|} by the following relation:

(7) $$\begin{array}{cc} F_{\text{ampl}}\left(U,V\right) & =1-\frac{1}{2N}\underset{ij}{\sum }{\left(\left|u_{ij}|-|v_{ij}\right|\right)}^{2}\\ & =1-\frac{1}{2N}\tau_{\text{ampl}}^{2}\left(U,V\right), \end{array}$$

where we have defined:

(8) $$\tau_{\text{ampl}}^{2}\left(U,V\right)=\underset{ij}{\sum }{\left(\left|u_{ij}|-|v_{ij}\right|\right)}^{2},$$

which is the amplitude total squared variation (TSV) calculated between *U* and *V*. The analytical proof of Eqn. (7) is reported in the Supplementary Materials (Section S2). Although the amplitude fidelity represents an easy way to evaluate the accuracy of a UPP, it is also easy to show that this figure of merit is flawed by a strong bias that reaches its minimum value as *N* approaches infinity. More specifically, in the Supplementary Materials (Section S2), we prove that:

(9) $$E\left[F_{\text{ampl}}\left(U,V\right)\right]∼\frac{\pi }{4}\;\text{ as }N\to \infty ,$$

where the operator *E*[⋅] is the expectation value of the amplitude fidelity calculated over Haar randomly distributed *U*, *V*. Besides this, the amplitude fidelity is also not suitable to evaluate the performance of a UPP in a multiphoton experiment, since in this case also the angles of the matrix elements play an important role.

### 5.2 Fidelity

Provided that a reconstruction of both amplitudes and angles of each complex matrix element is possible [24], the actual device fidelity F can be evaluated as follows:

(10) $$F\left(U,V\right)=\frac{1}{N}|tr\left(U^{†}V\right)|=\frac{1}{N}\underset{i,j}{\sum }u_{ij}^{†}v_{ij},$$

where we can remove the absolute value since *U* and *V* are always known up to a global phase term *e* ^*iψ*^ that can be arbitrarily chosen. As an example, a similar figure of merit was employed in [8] thanks to two-photon measurements allowing the reconstruction of the angles. The fidelity represents the normalized Frobenius inner product between *U* and *V*. Similarly to the amplitude fidelity, it is equal to 1 if and only if *U* = *V*, it is always included in the interval [0,1] and it is directly linked to the variation matrix *U* − *V* = {*u*_*ij*_ − *v*_*ij*_} by the following relation:

(11) $$\begin{array}{cc} F\left(U,V\right) & =1-\frac{1}{2N}\underset{ij}{\sum }{\left(u_{ij}-v_{ij}\right)}^{2}\\ & =1-\frac{1}{2N}\|U-V{\|}^{2}, \end{array}$$

where we have defined:

(12) $$\|U-V{\|}^{2}=\underset{ij}{\sum }{\left(u_{ij}-v_{ij}\right)}^{2},$$

in which ‖*U* − *V*‖ is the Frobenius norm calculated on the variation matrix *U* − *V*. The analytical proof of Equation (11) is reported in the Supplementary Materials (Section S2), along with the proof that the quantity ‖*U* − *V*‖^2^ is given by two separate contributions:

(13) $$\|U-V{\|}^{2}=\tau_{\text{ampl}}^{2}\left(U,V\right)+\tau_{\text{angle}}^{2}\left(U,V\right),$$

where we have defined:

(14) $$\tau_{\text{angle}}^{2}\left(U,V\right)=4\underset{ij}{\sum }|u_{ij}v_{ij}|\sin^{2}\frac{∠u_{ij}-∠v_{ij}}{2}.$$

The latter is the counterpart of the amplitude TSV, and we define it as the angle TSV. Wrapping up the discussion, we can conclude from Equations (11) and (13) that:

(15) $$F\left(U,V\right)=1-\frac{1}{2N}\left(\tau_{\text{ampl}}^{2}+\tau_{\text{angle}}^{2}\right).$$

From Equation (15) it is clear that, since it takes into account also the angle TSV, the fidelity F is always lower than the amplitude fidelity $F_{\text{ampl}}$ calculated on the same matrix pair *U*, *V*. Related to this, it is also worth noting that the fidelity F is a quasi-unbiased figure of merit, in the sense that the bias of the expectation value of the fidelity calculated on Haar randomly distributed unitary matrices *U*, *V* vanishes as *N* approaches infinity. More specifically, in the Supplementary Materials (Section S2), we prove that:

(16) $$E\left[F\left(U,V\right)\right]∼\frac{\sqrt{\pi }}{2N}\;\text{ as }N\to \infty .$$

### 5.3 Numerical simulation

Given the doubts raised on the amplitude fidelity, we decided to investigate how $F_{\text{ampl}}$ is related to F in the presence of errors. In detail, we used a Montecarlo simulator and phase errors attributed with a simplified stochastic model.

The simulator goes through the following steps:

1. Sample a random Haar unitary matrix *U*_set_ ∈ *U*(6).
2. Find the set of phases *θ*_*i*_ and *ϕ*_*i*_ corresponding to *U*_set_ using the decomposition algorithm reported in [7].
3. Introduce a random phase noise *ɛ* uniformly distributed in the interval *ɛ*_max_[−*π*, *π*] on both *θ*_*i*_ and *ϕ*_*i*_.
4. Get *U*_sim_ by matrix multiplication of each MZI layer.
5. Evaluate the effect of the noise by employing both the amplitude fidelity $F_{\text{ampl}}\left(U_{\text{set}},U_{\text{sim}}\right)$ and the actual fidelity $F\left(U_{\text{set}},U_{\text{sim}}\right)$ .

**Figure 7:**
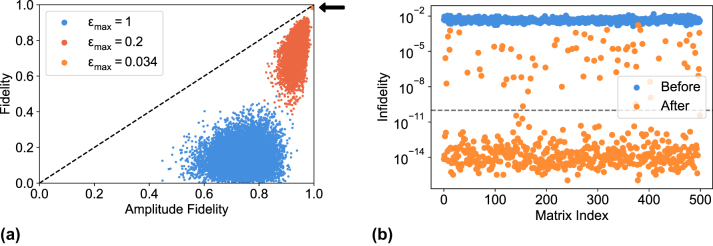
Simulated scatter plots of fidelity. (a) Scatter plot of the simulated fidelities for different values of the random phase noise *ɛ*_max_. The dashed line represents the upper bound of the plot, given by $F<F_{\text{ampl}}$ . The black arrow points to the data corresponding to *ɛ*_max_ = 0.034. (b) Scatter plot of the simulated fidelities after the optimization procedure was performed using the amplitude fidelity as a loss function. The dashed line represents the convergence threshold 1−F = 10^−10^.

Figure 7a reports the results of 10,000 iterations for three different values of the parameter *ɛ*_max_. For *ɛ*_max_ = 1, phases can be considered completely random. Nevertheless, an average amplitude fidelity $\bar{F_{\text{ampl}}}=0.7411$ is obtained, consistently with the bias that affects this figure of merit. On the contrary, the fidelity is a good witness of the high error affecting the phase set, since the simulation produces an average value $\bar{F}=0.1475$ . For *ɛ*_max_ = 0.2, errors are reduced and the amplitude fidelity steeply increases up to $\bar{F_{\text{ampl}}}=0.9399$ . However, the statistical dispersion remains quite large and many unitaries display values lower than 0.9, clearly indicating that something is not working in the processor. In fact, the fidelity remains on average $\bar{F}=0.7619$ , and several unitaries with very high amplitude fidelity $\left(F_{\text{ampl}}>0.95\right)$ have poor fidelity (F<0.6) . Interestingly, the situation looks completely different for *ɛ*_max_ = 0.034. This value was chosen to match the amplitude fidelity distribution measured on UPP A both in terms of average and standard deviation, i.e., $F_{\text{ampl}}=0.9978\pm 0.0008$ , compared to $F_{\text{ampl}}=0.9979\pm 0.0009$ as reported in Section 4.1. In this case, points are all concentrated in a tight spot at the top right corner of the graph in Figure 7a; with this phase noise, the fidelity is as high as F=0.9921±0.0029 , which is very close to the amplitude fidelity. This suggests that a low statistical dispersion of the amplitude fidelity, around a high value close to 1, is likely to be associated to low errors also on the angles.

We note that, while our model assumed uniform distributions of the phase errors around the correct values, by studying this simple case, we are gaining clues on a more general case. If we considered errors in phases with different shapes of the statistical distributions, the “clouds” of points that would be produced in the graph of Figure 7a would be actually a subset of a larger “cloud” of points produced by our Montecarlo simulator, provided that the error dispersion *ɛ*_max_ is properly chosen. Even imperfections in the fabrication of the unit cell (such as an imperfect reflectivity of the couplers) could be translated to phase errors with a proper distribution, as Equation (1) is a sufficiently general expression for a 2 × 2 unitary matrix, parameterized with the phases *θ* and *ϕ*.

It should finally be noted that it is generally easy to find unitary matrices with $F_{\text{ampl}}=1$ and F arbitrarily low, if one is able to act at will on the complex entries of the matrix, keeping only the constraint of unitarity. However, in our experiments, there is not such a freedom. We are indeed acting on a specific decomposition of the original unitary matrix: the phase terms in our circuit are calculated analytically taking into account all moduli and angles of the elements of the desired unitary matrix. Each of the phase terms affects nontrivially many of the matrix elements at the same time. Thus, it is not too unreasonable that small changes around the ideally correct values (whichever their probability distribution) have action both on the amplitudes and on the angles of the matrix elements. As suggested by our simulations, F and $F_{\text{ampl}}$ are likely to decrease simultaneously or, conversely, if a high $F_{\text{ampl}}$ is retained, this can be associated to a high value of F . These observations strengthen the validity of the experimental characterization performed on our UPPs.

As a second step, one could also put into question the choice of the amplitude fidelity as a loss function for the optimization process discussed in Section 4.2. Therefore, we decided to modify the simulator in order to implement the following procedure:

1. Sample a random Haar unitary matrix *U*_set_ ∈ *U*(6).
2. Find a set of phases *θ*_*i*_ and *ϕ*_*i*_ corresponding to *U*_set_ using the decomposition algorithm reported in [7].
3. Introduce a random phase noise *ɛ* suitably distributed to match average and standard deviation of the amplitude fidelity distribution measured for UPP A (Figure 7a, orange dots).
4. Apply the minimization algorithm by calculating *U*_sim_ by matrix multiplication of each MZI layer and by using the amplitude infidelity $1-F_{\text{ampl}}\left(U_{\text{set}},U_{\text{sim}}\right)$ as a loss function.
5. Evaluate the final effect of the optimization algorithm by employing the actual infidelity $1-F\left(U_{\text{set}},U_{\text{sim}}\right)$ .

Figure 7b shows the results of 500 iterations of this algorithm, reporting the optimization in terms of infidelity 1−F . Despite being based on the amplitude fidelity as the loss function, the algorithm led to a remarkable improvement of the fidelity, with the 86 % of the matrices reaching full convergence (arbitrarily defined as 1−F < 10^−10^, dashed line in Figure 7b). Indeed it is worth noting that no matrix showed a fidelity worse than the initial condition, demonstrating the effectiveness of our optimization protocol based only on intensity measurements.

## 6 Conclusions

In this work, we reported on the design, fabrication, and characterization of two 6-mode UPPs fabricated in a FLW integrated photonic platform. These devices find their natural application in quantum optics and quantum information experiments. The advantages of our technology for this set of applications are manifold. First of all, they are compatible with quantum sources emitting both in the visible range and at telecom wavelength (here demonstrated at 785 nm and 1550 nm) with no penalty in terms of photon losses. Secondly, the high precision reached with our calibration protocol allows for the implementation of arbitrary optical transformations with average fidelity higher than 0.9970, which can be pushed over 0.9990 thanks to an optimization algorithm based only on intensity measurements. Last, the low insertion losses (<3 dB) make them also compatible with state-of-the-art multiphoton experiments.

This work fits into a broad literature on this topic that we can summarize as follows. Focusing on the FLW platform, larger processors have been already reported in the literature [18], [26]. However, they either do not explicitly show a reconfigurability as large as ours [18] or lack the capability to control the device reconfiguration [26]. Therefore, we can conclude that our UPPs provide the highest level of reconfigurability and control demonstrated to date in a FLW platform, with the additional feature of reporting polarization-insensitive optical transformations. Then, if we take into account also the other fabrication platforms, the comparison of our FLW-UPPs with the silicon nitride ones arises spontaneously. These devices have been reported both at 940 nm [10] and 1550 nm [9]. The authors provide a thorough characterization of their platform, reporting an average amplitude fidelity of 0.986 and 0.974, respectively, for the implementation of random Haar transformations. In absolute terms, these numbers are lower with respect to ours. It needs, however, to be stated that these UPPs feature 12 and 20 modes, respectively, and an easy way to make a fair comparison between processors with a different level of complexity does not exist at the moment. On the other hand, our FLW-UPPs are able to manipulate different polarization states of light, a feature that has never been reported in silicon nitride, and our FLW programmable MZIs are characterized by a power dissipation of about one order of magnitude lower (i.e., tens vs. hundreds of milliwatts), which is of key importance for the scalability.

In the future, we believe that scaling the number of modes to a level comparable to the one already demonstrated for the silicon nitride platform will be the main focus of the research on FLW-UPPs. In this regard, the limited power dissipation of FLW programmable MZIs, combined with the next generation of curved isolation structures [27], will be the key for scaling toward tens of modes with limited cost in terms of circuit length and losses, thus unlocking a new level of complexity for high-fidelity polarization-insensitive UPPs.

## Supplementary Material

Supplementary Material Details
