# Supplementary material for: High-fidelity and polarization-insensitive universal photonic processors fabricated by femtosecond laser writing
Source: Nanophotonics. 2024 Jan 16;13(12):2259–70. doi: 10.1515/nanoph-2023-0636 (PMC11501604; doi:10.1515/nanoph-2023-0636)
Supplement: Supplementary file 1 — Supplementary Material Details [file j_nanoph-2023-0636_suppl_001.pdf]

Supplementary Materials: High-fidelity and polarization-insensitive universal photonic processors fabricated by femtosecond laser writing

Ciro Pentangelo[1,2], Niki Di Giano[1,2], Simone Piacentini[2], Riccardo Arpe[1], Francesco Ceccarelli[2,1], Andrea Crespi[1,2], and Roberto Osellame[2,1]

[1]Dipartimento di Fisica, Politecnico di Milano, Milano, Italy
[2]Istituto di Fotonica e Nanotecnologie, Consiglio Nazionale delle Ricerche (IFN-CNR), Milano, Italy

S1 Operation of the UPPs with coherent light input

In this section we discuss the experimental apparatus used for the calibration and operation of the two devices (Figure 1), as well as the setup used for the polarization measurements (Figure 2).

For UPP A, a 785 nm laser diode was used (Thorlabs L785P25), controlled by a Thorlabs Kinesis K-Cube laser diode driver; the laser temperature was controlled and kept stable using a Thorlabs T-Cube TEC controller. The elliptical laser beam profile was corrected with an anamorphic prism pair. For UPP B, a 1550 nm laser diode (Thorlabs L1550P5DFB) was used, controlled by an equivalent driver.

After being steered in air through mirrors, the laser beam was coupled into a single-mode fiber using an aspheric lens (NA = 0.15). This fiber was connected to the input of an optical fiber switch (LFiber), in turn connected to a fiber array. Light from the output ports of the devices was collected by a second fiber array and routed to a power meter (Thorlabs PM16-120/122) via a second fiber switch (LFiber). Different fiber switches were employed for the two processors UPP A and UPP B, due to the different operation wavelengths. The fiber switches can be remotely controlled by a computer via a Python script. In order to characterize the implemented unitary matrices the coherent laser light was routed in order into each input port of the UPP, and the optical power from the output ports was sequentially measured.

The current flowing through each thermal shifter was controlled using a 32-channel computer-controlled power supply (Qontrol). Each channel can provide up to 24 mA current or 12 V volts, with a 16-bit precision (±370 nA, ±180 µV). Temperature stabilization of the circuit was performed by mounting the UPP on an aluminum heat sink, equipped with a Peltier cell. The electrical current

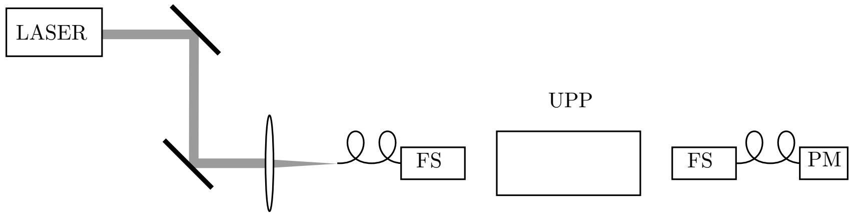

Figure 1: Experimental setup for the calibration and operation of the devices with arbitrary polarization. FS = fiber switch, UPP = universal photonic processor, PM = power meter.

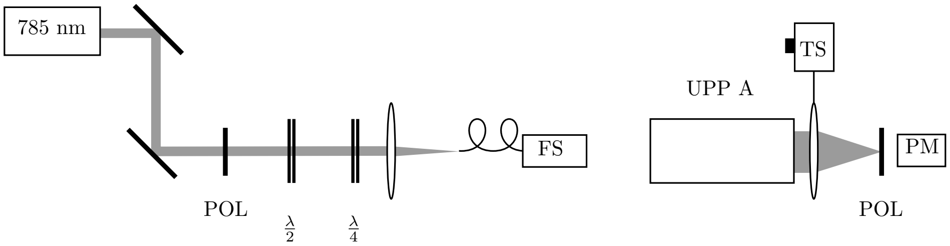

Figure 2: Experimental setup for the characterization of the behavior of UPP A with polarized light. POL = polarizer, FS = fiber switch, UPP = universal photonic processor, TS = translation stage, PM = power meter.

flowing through the Peltier cell was regulated in closed loop by a proportional-integral-derivative (PID) controller, fed by a temperature sensor fixed to the aluminum sink. The temperature of the devices has been kept stable at 20 °C for the entire calibration and operation processes.

The setup used to perform the polarization measurements (Figure 2) presents the same basic structure as the calibration setup. To allow the selection of a specific polarization state at the input of the UPP, a sequence of a polarizer, a half-wave plate and a quarter-wave plate was added before the aspheric lens of the fiber-coupling stage. These three components are mounted in suitable rotation mounts, and enable us to compensate for polarization rotations occurring in the input fibers. Moreover, to remove additional and uncontrolled polarization rotations at the output of the device, the fiber array at the output of the device was removed and replaced with an aspheric lens (NA = 0.68), mounted on a motorized translation stage, that collects the light and images the desired output port on the power meter head. A polarizer, also mounted on a rotation mount, was further added before the power meter head.

The polarization state right at the output of the laser diode is approximately linear, oriented at about 45° with respect to the plane of the optical bench, the latter being understood as the reference horizontal (H) direction. An initial setting of the apparatus is operated by rotating the input polarizer to the vertical (V) position and the output polarizer to the H position, and by tuning the angles of the half-wave and quarter-wave plates until the power measured is minimized. Since our waveguides possess a birefringence axis oriented vertically [1], this configuration implies that the light propagating in the UPP is linearly polarized with V orientation. To perform the actual measurements on the UPP with V polarization, we just rotated back the output polarizer to the V position. Instead, to perform the measurements with H polarization, starting from the same initial

setting we rotated the input polarizer to the H position.

S2 Proofs of Mathematical Claims

Theorem 1. *Let $V, U \in U(N)$ be unitary matrices, randomly and independently distributed according to the Haar measure. Then, the expected value of the fidelity $\mathcal{F}(V,U)$, averaged over the entire space of the possible U and V, in the limit $N \to \infty$ is asymptotic to $\frac{\sqrt{\pi}}{2N}$. Namely:*

$$E[\mathcal{F}(V,U)] = \iint_{U(N)\times U(N)} \mathcal{F}(V,U)\,\mathrm{d}\mu(U)\mathrm{d}\mu(V) \;\sim\; \frac{\sqrt{\pi}}{2N} \tag{1}$$

where $\mathrm{d}\mu(\cdot)$ is the Haar measure and $\mathcal{F}(V,U) = \frac{1}{N}\left|\mathrm{tr}\big(V^{\dagger}U\big)\right|$.

Proof. We begin by fixing one random V and by evaluating the average $\mathcal{F}(V,U)$ on the space of the U:

$$\int_{U(N)} \mathcal{F}(V,U)\mathrm{d}\mu(U) = \int_{U(N)} \frac{1}{N}\left|\mathrm{tr}\big(V^{\dagger}U\big)\right| \mathrm{d}\mu(U). \tag{2}$$

By exploiting the defining property of the measure,

$$\mathrm{d}\mu(U) = \mathrm{d}\mu(V^{\dagger}U) \tag{3}$$

we operate a change of variables $V^{\dagger}U = Z$. The integral (2) now reads

$$\frac{1}{N}\int_{U(N)} |\mathrm{tr}(Z)|\,\mathrm{d}\mu(Z). \tag{4}$$

It was proven by Diaconis and Shahshahani [2] that in the limit $N \to \infty$, the probability distribution of the trace of a unitary matrix approaches that of a complex standard normal random variable. Therefore:

$$\int_{U(N)} |\mathrm{tr}(Z)|\,\mathrm{d}\mu(Z) \to \int_{-\infty}^{\infty}\int_{-\infty}^{\infty} |x+iy|\,\rho(x,y)\mathrm{d}x\mathrm{d}y, \tag{5}$$

where ρ is the probability density of a complex standard normal random variable in terms of the real and imaginary parts x and y, i.e. a pair of independent normally distributed real random variables with variance 1/2. We can write it explicitly:

$$\rho(x,y) = \frac{1}{\sqrt{\pi}}e^{-x^2}\cdot\frac{1}{\sqrt{\pi}}e^{-y^2} \tag{6}$$

and therefore calculate:

$$\int_{U(N)} |\mathrm{tr}(Z)|\,\mathrm{d}\mu(Z) \to \frac{1}{\pi}\int_{-\infty}^{\infty}\int_{-\infty}^{\infty} \sqrt{x^2+y^2}\,e^{-(x^2+y^2)}\mathrm{d}x\mathrm{d}y = \Gamma\left(\frac{3}{2}\right) = \frac{1}{2}\sqrt{\pi}. \tag{7}$$

We thus conclude:

$$\frac{1}{N}\int_{U(N)} \left|\mathrm{tr}\big(V^{\dagger}U\big)\right| \mathrm{d}\mu(U) \sim \frac{\sqrt{\pi}}{2N} \quad \text{for} \quad N \to \infty. \tag{8}$$

This value does not depend on V, thus averaging further on the space of the V we get this same value and we have proven the Theorem. $\square$

Theorem 2. *Let $V, U \in U(N)$ be unitary matrices, randomly and independently distributed according to the Haar measure. Then, the expected value of the amplitude fidelity $\mathcal{F}_{\text{ampl}}(V,U)$, averaged over the entire space of the possible U and V, in the limit $N \to \infty$ is asymptotic to $\frac{\pi}{4}$. Namely:*

$$E[\mathcal{F}_{\text{ampl}}(V,U)] = \iint_{U(N)\times U(N)} \mathcal{F}_{\text{ampl}}(V,U)\,\mathrm{d}\mu(U)\mathrm{d}\mu(V) \;\sim\; \frac{\pi}{4} \tag{9}$$

where $\mathrm{d}\mu$ is the Haar measure and $\mathcal{F}_{\text{ampl}}(V,U) = \dfrac{1}{N}\operatorname{tr}\big(|V^\dagger||U|\big)$. In the latter expression, if $A = \{a_{ij}\}$ we are indicating as $|A|$ the matrix $|A| = \{|a_{ij}|\}$.

Proof. Consider the following expression:

$$E[\mathcal{F}_{\text{ampl}}(V,U)] = E\left[\frac{1}{N}\operatorname{tr}\big(|V^\dagger||U|\big)\right] = E\left[\frac{1}{N}\sum_{ij}|v_{ij}||u_{ij}|\right] = NE\left[\frac{1}{N^2}\sum_{ij}|v_{ij}||u_{ij}|\right]. \tag{10}$$

The expression inside the expectation value brackets $\frac{1}{N^2}\sum_{ij}|v_{ij}||u_{ij}|$ is actually equal to the average value of the product $|v_{ij}||u_{ij}|$ over all the values of the indices i, j, i.e. over all the matrix entries. The entries u_{ij} and v_{ij} are independent but identically distributed random variables. Therefore, as $N \to \infty$,

$$E[\mathcal{F}_{\text{ampl}}] \sim NE[E[|v_{ij}||u_{ij}|]] = NE[|v_{ij}||u_{ij}|] = NE[|v_{ij}|]E[|u_{ij}|] = NE[|u_{ij}|]^2. \tag{11}$$

Since any entry of a unitary matrix as $N \to \infty$ is approximately a complex normal random variable with variance N^{-1} (see for example [3] pag. 141), we have

$$E[|u_{ij}|] = \frac{N}{\pi}\int_{-\infty}^{\infty}\int_{-\infty}^{\infty}\sqrt{x^2+y^2}\,e^{-N(x^2+y^2)}\mathrm{d}x\mathrm{d}y \tag{12}$$

and we can reduce this integral to the integral in Equation 7 (apart from constant factors) with a change of variables. At the end, we obtain

$$E[|u_{ij}|] = \sqrt{\frac{1}{N}}\Gamma\left(\frac{3}{2}\right) = \sqrt{\frac{\pi}{4N}} \tag{13}$$

and therefore, for $N \to \infty$ the amplitude fidelity approaches a constant asymptote:

$$E[\mathcal{F}_{\text{ampl}}] \sim \frac{\pi}{4}. \tag{14}$$

$\square$

Theorem 3. *With the definition of the amplitude fidelity $\mathcal{F}_{\mathrm{ampl}}(V,U)$ as in Theorem 2, it holds*

$$\mathcal{F}_{\mathrm{ampl}}(V,U) = 1 - \frac{1}{2N}\sum_{ij}(|v_{ij}| - |u_{ij}|)^2. \tag{15}$$

Proof. Consider the Frobenius norm of the difference $|V| - |U|$:

$$\begin{aligned} \||V| - |U|\|^2 &= \mathrm{tr}\big((|V| - |U|)^\dagger(|V| - |U|)\big) = \\ &= \mathrm{tr}\big(|V^\dagger||V| + |U^\dagger||U| - |V^\dagger||U| - |U^\dagger||V|\big) = \\ &= \mathrm{tr}\big(|V^\dagger||V|\big) + \mathrm{tr}\big(|U^\dagger||U|\big) - \mathrm{tr}\big(|V^\dagger||U|\big) - \mathrm{tr}\big(|U^\dagger||V|\big) = \\ &= \|V\|^2 + \|U\|^2 - 2\,\mathrm{tr}\big(|V^\dagger||U|\big). \end{aligned} \tag{16}$$

Since V and U are both unitary, $\|V\|^2 = \|U\|^2 = N$. The square of the Frobenius norm of a matrix A is also equal to the sum of the squares of every entry of the matrix. Rearranging the above expression yields

$$\begin{aligned} \mathrm{tr}\big(|V^\dagger||U|\big) &= N - \frac{1}{2}\||V| - |U|\|^2 = \\ &= N - \frac{1}{2}\sum_{ij}(|v_{ij}| - |u_{ij}|)^2. \end{aligned} \tag{17}$$

Therefore, using the definition as given in Theorem 2,

$$\begin{aligned} \mathcal{F}_{\mathrm{ampl}}(V,U) &= \frac{1}{N}\,\mathrm{tr}\big(|V|^\dagger|U|\big) = \\ &= 1 - \frac{1}{2N}\sum_{ij}(|v_{ij}| - |u_{ij}|)^2 \end{aligned} \tag{18}$$

as required. □

Theorem 4. *With the definition of fidelity $\mathcal{F}(V,U)$ as in Theorem 1, up to a global phase term that multiplies V or U,*

$$\mathcal{F}(V,U) = 1 - \frac{1}{2N}\sum_{ij}|v_{ij} - u_{ij}|^2. \tag{19}$$

Moreover following the definition of amplitude fidelity $\mathcal{F}_{\mathrm{ampl}}(V,U)$ as given in Theorem 2,

$$\mathcal{F}(V,U) = \mathcal{F}_{\mathrm{ampl}}(V,U) - \frac{2}{N}\sum_{ij}|v_{ij}u_{ij}|\sin^2\left(\frac{\angle u_{ij} - \angle v_{ij}}{2}\right), \tag{20}$$

where $\angle v_{ij}, \angle u_{ij}$ are the phases of v_{ij}, u_{ij} respectively.

Proof. The first part of the proof follows closely the proof of Theorem 3. In fact, the same calculation shows that

$$
\begin{aligned}
\|V-U\|^2 &= \operatorname{tr}\big((V-U)^\dagger(V-U)\big) = \\
&= \operatorname{tr}\big(V^\dagger V + U^\dagger U - V^\dagger U - U^\dagger V\big) = \\
&= \operatorname{tr}\big(V^\dagger V\big) + \operatorname{tr}\big(U^\dagger U\big) - \operatorname{tr}\big(V^\dagger U\big) - \operatorname{tr}\big(U^\dagger V\big) = \\
&= \|V\|^2 + \|U\|^2 - 2\operatorname{Re}[\operatorname{tr}\big(V^\dagger U\big)] = \\
&= 2N - 2\operatorname{Re}[\operatorname{tr}\big(V^\dagger U\big)].
\end{aligned}
\tag{21}
$$

Since the choice of global phase is arbitrary, we can always choose to multiply U or V by a global phase such that $\operatorname{tr}\big(V^\dagger U\big)$ is real and positive. With such condition we have

$$
\operatorname{Re}[\operatorname{tr}\big(V^\dagger U\big)] = \left|\operatorname{tr}\big(V^\dagger U\big)\right|. \tag{22}
$$

Moreover,

$$
\|V-U\|^2 = \sum_{ij} |v_{ij} - u_{ij}|^2 ; \tag{23}
$$

plugging this into the definition of fidelity from Theorem 1 the first result follows:

$$
\mathcal{F}(V,U) = 1 - \frac{1}{2N}\sum_{ij} |v_{ij} - u_{ij}|^2 . \tag{24}
$$

To prove the second statement, if we introduce the phases $\alpha_{ij} = \angle u_{ij}$ and $\beta_{ij} = \angle v_{ij}$ we can rewrite

$$
\begin{aligned}
|v_{ij} - u_{ij}|^2 &= \left| |v_{ij}| e^{i\beta_{ij}} - |u_{ij}| e^{i\alpha_{ij}} \right|^2 = \\
&= \left| |v_{ij}| - |u_{ij}| e^{i(\alpha_{ij}-\beta_{ij})} \right|^2 = \\
&= |v_{ij}|^2 + |u_{ij}|^2 - 2|v_{ij}u_{ij}|\cos(\alpha_{ij}-\beta_{ij}) = \\
&= |v_{ij}|^2 + |u_{ij}|^2 - 2|v_{ij}u_{ij}| + 2|v_{ij}u_{ij}|(1-\cos(\alpha_{ij}-\beta_{ij})) = \\
&= (|v_{ij}| - |u_{ij}|)^2 + 2|v_{ij}u_{ij}|(1-\cos(\alpha_{ij}-\beta_{ij})) = \\
&= (|v_{ij}| - |u_{ij}|)^2 + 4|v_{ij}u_{ij}|\sin^2\left(\frac{\alpha_{ij}-\beta_{ij}}{2}\right).
\end{aligned}
\tag{25}
$$

This implies that

$$
\mathcal{F}(V,U) = 1 - \frac{1}{2N}\sum_{ij} (|v_{ij}| - |u_{ij}|)^2 - \frac{1}{2N}\sum_{ij} 4|v_{ij}u_{ij}|\sin^2\left(\frac{\alpha_{ij}-\beta_{ij}}{2}\right) \tag{26}
$$

and using the result of Theorem 3 we conclude:

$$
\mathcal{F}(V,U) = \mathcal{F}_{\text{ampl}}(V,U) - \frac{2}{N}\sum_{ij} |v_{ij}u_{ij}|\sin^2\left(\frac{\alpha_{ij}-\beta_{ij}}{2}\right). \tag{27}
$$

□

References

[1] Giacomo Corrielli, Simone Atzeni, Simone Piacentini, Ioannis Pitsios, Andrea Crespi, and Roberto Osellame. Symmetric polarization-insensitive directional couplers fabricated by femtosecond laser writing. *Optics Express*, 26(12):15101–15109, 2018.

[2] Persi Diaconis and Mehrdad Shahshahani. On the eigenvalues of random matrices. *Journal of Applied Probability*, 31:49–62, 1994.

[3] Fumio Hiai and Denes Petz. *The semicircle law, free random variables and entropy.* American Mathematical Society, 2006.
